# 

**Published:** 1853-03

**Authors:** 


					﻿THE
WESTERN JOURNAL*
OF
MEDICINE AND SURGERY.
EDITORS.
LUNSFORD P. YANDELL, M. D.
PROFESSOR OF PHYSIOLOGY AND PATHOLOGICAL ANATOMY
IN THE UNIVERSITY OF LOUISVILLE.
ABD
THEODORE S. BELL, M.D.
Receipts of Medical Journal for February and March, 1853.
Dr. E. C. Colvard, Shawneetown, Ill.,	-	-	*	85	00
Dr. R. S. Newton. Cincinnati, Ohio,	-	-	-	10	00
Dr. W. J. Routh, Berrysville, Ark.,	-	-	-	5	00
Dr. Kerrick, Lacona. Ky., -	-	-	-	-	10	CO
Dr. L. W. Wilson, Centreville, Ala.,	-	-	-	10	00
Dr. G. W. Booth, Carrollville, Miss.,	-	-	-	10	00
Dr. J. S- Croutch, Clarksville, Tenn.,	-	-	-	10	00
Dr. R. Searcy, Tuscaloosa, Ala.,	-	-	-	-	15	00
Dr. J. Doran, Hodgenville, Ky.,	-	-	-	-	5	00
Dr. W. Miller, Terre Haute, Ind.,	-	-	• -	5 00
Dr. C.	B. Sullivan, Waterloo, Ala., -	-	-	-	10	00
Dr. D.	E. Gibson, Bellemont, Tenn.,	-	t	-	-	10	00
Dr. N.	K. Moxley, Wheelersburg, Ohio,	-	*	-	-	5	00
Dr. C. Carli, Still Water Lake, Minnesota,	-	-	5 00
Dr. T.	N. Shelby, Port Gibson, Miss ,	-	-	-	5	00
Drs. Throop & Still, Head Quarters, Ky.,	-	-	-	5	00
Di^ W. B. Barfield, Linden, Tenn., -	-	-	-	5	00
Di* A. A. Bennett, Oxford, Ohio, -	-	-	-	5	00
Drs. Grimes <Sc Henry, Hopkinsville, Ky.,	-	-	-	3	00
Dr. T. H. Stovall, Middleton, Miss.,	-	-	5 00
Dr. T. Lipscomb, Shelbyville, Ky., -	-	-	5 00
Dr. J. W. Embree, Belton, Texas, -	-	-	5 00
Dr. E. A. Rony, Athens, Ky.,	-	.	-	-	5	00
Dr. F. P. Alford, Aurora, Ky.	-	-	-	-	5	00
Dr. W. Brice, Halselville, S. C.,	-	-	-	-	5	00
Dr. S. W. B. McClurkin, Halselvill., S. C.,	-	5 00
The Westers Journal of Medicine and Surgery is published monthly by
the undersigued, at the comer of ^tiin and Fifth streets, Louisville, at $5 per
annum, payable in advance. Each number contains 96 pages, making two vol-
umes m the year of more than 550 pages each.
Contributions to its pages by the Physicians of the Valley of the Mississippi
addressed to the Editors, are respectfully solicited.
Letters on business, to De addressed,postage paid, to the publishers.
PRENTICE HENDERSON
				

